# Postprandial plasma aminoacidemia and indices of appetite regulation following pea-rice blend, pea isolate and whey protein ingestion in healthy young adults

**DOI:** 10.1017/S0007114524001958

**Published:** 2024-09-28

**Authors:** Lucy M. Rogers, Archie E. Belfield, Marie Korzepa, Ari Gritsas, Tyler A. Churchward-Venne, Leigh Breen

**Affiliations:** 1 School of Sport, Exercise and Rehabilitation Sciences, University of Birmingham, Birmingham B15 2TT, UK; 2 MRC-Versus Arthritis Centre for Musculoskeletal Ageing Research, University of Birmingham, Birmingham, UK; 3 Research Institute of the McGill University Health Centre, Montreal, QC, Canada; 4 Department of Kinesiology and Physical Education, McGill University, Montreal, QC, Canada; 5 Division of Geriatric Medicine, McGill University, Montreal, QC, Canada; 6 NIHR Biomedical Research Centre, Birmingham, UK

**Keywords:** Plant-blend protein, Bioavailability, Appetite, Energy intake

## Abstract

Plant-derived proteins are often deficient in essential amino acids and have lower rates of digestibility than animal-derived proteins. Blending different plant-derived proteins could compensate for these deficiencies and may augment postprandial aminoacidemia over single-source plant proteins. This study assessed plasma amino acids and appetite hormones, appetite sensations and *ad libitum* energy intake following ingestion of a pea-rice protein blend (BLEND), compared with pea-only (PEA) and whey (WHEY) protein. In a randomised, double-blind, crossover design, ten healthy adults (M *n* 4, F *n* 6; mean (sd) age 22 (sd 3) years; BMI 24 (sd 3) kg·m^2^) ingested 0·3 g·kg·body mass^–1^ of BLEND, PEA or WHEY. Arterialised venous blood samples and appetite ratings were obtained in the fasted state and over 240 min postprandially. Energy intake was measured via an *ad libitum* buffet-style test meal. Mean plasma essential amino acid incremental AUC was higher in WHEY, compared with PEA (*P* < 0·01; mean diff (95 % CI): 44 218 (15 806, 72 631) μmol·240 min·l^–1^) and BLEND (*P* < 0·01; 14 358 (16 031, 101 121) μmol·240 min·l^–1^), with no differences between PEA and BLEND (*P* = 0·67). Plasma ghrelin and glucagon-like peptide-1, appetite ratings and a*d libitum* energy intake responses did not differ between treatments (*P* > 0·05 for all). Ingestion of a pea-rice protein blend did not augment postprandial aminoacidemia above pea protein, perhaps attributable to marginal differences in essential amino acid composition. No between-treatment differences in appetite or energy intake responses were apparent, suggesting that the influence of protein ingestion on perceived appetite ratings and orexigenic hormonal responses may not be solely determined by postprandial plasma aminoacidemia.

Protein nutrition increases rates of muscle protein synthesis^(1)^ through the postprandial rise in circulating essential amino acid (EAA) concentrations^(2,3)^, particularly leucine^(4,5)^. Importantly, plasma aminoacidemia following protein ingestion is contingent on the constituent amino acid content and the digestion and absorption kinetics of a protein source^(6)^. These characteristics primarily determine the quality of a protein source, suggested to have implications for muscle anabolism and remodelling^(7–10)^.

Typically, animal-derived proteins are deemed to be higher quality than plant-derived proteins. Indeed, for a given protein dose, most plant-derived sources exhibit a lower content of EAA and leucine^(11)^. Plant-derived proteins are also often deficient in one or more EAA, usually lysine and/or methionine^(11)^, and are thought to be more slowly digested and absorbed compared with animal-derived proteins^(12–15)^. Numerous studies^(10,16)^ have reported a smaller rise in circulating EAA concentrations following ingestion of plant-derived protein isolates compared with animal-derived protein isolates. For example, the plasma EAA response over 300 min following ingestion of 30 g of milk protein concentrate was 110 % greater than that of 30 g of wheat protein hydrolysate^(17)^. Similarly, ingestion of 30 g of potato protein concentrate resulted in a 16 % lower plasma EAA response over 300 min compared with 30 g of milk protein concentrate^(18)^. In both of these studies, the acute postprandial muscle protein synthesis responses were equivalent. This suggests that, beyond a certain point, a greater amplitude in postprandial plasma aminoacidemia may be negligible for postprandial muscle protein synthesis. Notwithstanding, nutritional strategies to bolster postprandial aminoacidemia in response to single-source plant-derived proteins hold the potential to optimise muscle anabolism, with important implications for muscle adaptive remodelling in the face of changing consumer behaviour around plant and animal protein consumption^(11,19)^.

Blending different plant-derived proteins with complementary EAA profiles could compensate for the specific deficiencies in single-source plant-derived proteins, thereby augmenting postprandial plasma aminoacidemia^(20)^. For example, combining brown rice protein (high in methionine but low in lysine) and pea protein (low in methionine but high in lysine) may yield a pea-rice protein blend with a balanced amino acid profile, void of any deficiencies^(11,20)^. However, few studies to date have investigated the postprandial amino acid response to such plant-blend proteins. Recently, Pinckaers and colleagues^(21)^ reported plasma EAA availability over a 300-min postprandial phase was 2-fold greater following ingestion of 30 g milk protein concentrate compared with a plant protein blend of 15 g wheat hydrolysate, 7·5 g corn isolate and 7·5 g of pea concentrate. Similarly, van der Heijden *et al.*^(22)^ recently demonstrated a 44 % greater 240 min plasma EAA availability following ingestion of 32 g of whey protein, compared with a dose-matched pea-rice-canola protein blend. Whether the ingestion of plant-blend proteins elicits a greater postprandial rise in circulating EAA compared with single-source plant-derived proteins remains to be investigated.

The rise in circulating amino acids following protein feeding may also have implications for satiety and the control of food intake. The aminostatic theory of food intake regulation^(23)^ proposes that reductions in appetite (and subsequent increases in satiety) following protein ingestion are related to increased blood availability of amino acids. As such, numerous studies have investigated the influence of different sources of dietary protein on indices of appetite regulation, with equivocal findings^(24–27)^. Indeed, the source of dietary protein may impact perceived sensations of appetite and/or satiety but bares little influence on *ad libitum* energy intake responses^(28,29)^. Conversely, very few studies to date have compared the appetite-regulatory hormonal responses to the ingestion of different sources of dietary protein. Notwithstanding the apparent discordance between plasma concentrations of gut-derived appetite hormones and energy intake in humans^(30)^, the assessment of several indices of appetite regulation is warranted to fully elucidate how divergent sources of protein may influence appetite. Indeed, recent research has called for a more extensive exploration of the potential mediators of protein-induced satiety^(29)^. Further, whilst numerous comparisons between the appetite-regulatory effects of plant- and animal-derived proteins exist^(29,31)^, there are no comparisons between plant-blend and single-source plant-derived proteins at present.

Therefore, the primary aim of the present study was to determine the postprandial change in plasma TAA, EAA, leucine, methionine and lysine following the ingestion of 0·3 g·kg·body mass^–1^ of a pea-rice protein blend, compared with a pea protein isolate and whey protein concentrate. Second, we aimed to determine plasma insulin, glucose and appetite-regulatory hormone concentrations, perceived appetite sensations and *ad libitum* energy intake responses to these divergent protein sources. We hypothesised that plasma EAA availability following ingestion of a pea-rice protein blend would be greater compared with pea protein isolate but lower compared with whey protein concentrate. In contrast, we theorised that postprandial appetite sensations, gut-derived hormone concentrations and *ad libitum* meal energy intake would not differ between protein beverages.

## Methods

### Participants

Four male and six female young healthy individuals volunteered to participate in this study (participant characteristics are presented in Table 1). Briefly, prospective participants were excluded based on the following criteria: aged < 18 or > 40 years, BMI < 18·5 or > 29·9 kg·m^2^, metabolic or respiratory disease or chronic illness, habitual smoker, known allergies or intolerances to study materials and supplements and the use of medications known to affect appetite or protein metabolism. Participants were informed of the primary study purpose (investigating postprandial aminoacidemia in response to ingestion of different protein isolates), experimental procedures and potential risks associated with participating before they provided written informed consent. Participants were not informed that *ad libitum* energy intake would be assessed as part of this study since knowledge of this measurement may have influenced dietary behaviours. Ethical approval was obtained by the Science, Technology, Engineering and Mathematics Ethical Review Committee at the University of Birmingham (ERN_21-1508), and all procedures were conducted in accordance with the Declaration of Helsinki (7th Edition).

**Table 1.** Participant characteristics

	Mean	sd
Age (years)	22	3
Mass (kg)	68·0	16·0
Height (m)	1·7	0·1
BMI (kg·m^–2^)	23·8	2·5
Fat mass (kg)	14·6	4·1
Fat-free mass (kg)	51·9	13·9
Body fat %	22·2	4·4

Data presented as mean ± sd; *n* 10.

### Study design

**Fig. 1.**
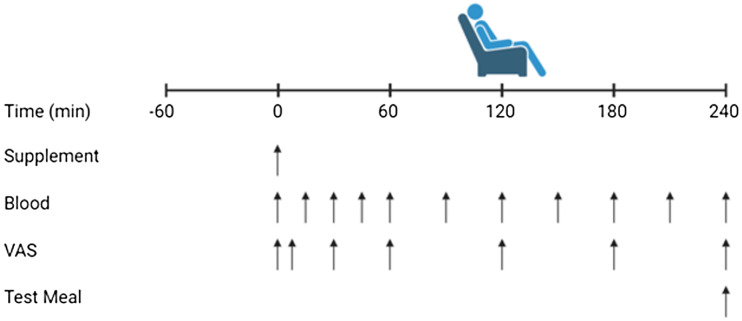
Schematic of study design. Trials were separated by > 5 d and involved ingestion of 0·3 g·kg·body mass^–1^ of a pea-rice protein blend (BLEND), pea protein (PEA) and whey protein (WHEY), arterialised venous blood sampling over 4 h and a buffet-style test meal for the assessment of *ad libitum* energy intake.

The present study followed a randomised, double-blind crossover design where participants completed one preliminary visit and three experimental trials at the University of Birmingham’s School of Sport, Exercise and Rehabilitation Science laboratories. The preliminary visit was conducted at least 1 week prior to the first experimental trial and involved eligibility screening, height and mass measurements and completion of a general health history questionnaire. For each experimental trial, separated by at least 5 d, participants were asked to ingest 0·3 g·kg·body mass^–1^ of pea-rice protein blend (BLEND), pea protein isolate (PEA) or whey protein concentrate (WHEY). Trial order was randomised and counterbalanced between participants to reduce any effect of trial order on study outcomes. Two independent researchers external to the research team were responsible for the trial allocation and preparation of supplemental beverages. Following supplement ingestion, participants rested in the laboratory for repeat blood sampling and appetite sensation measurements over 4 h, before consuming an *ad libitum* test meal (Fig. 1).

### Diet and physical activity

Twenty-four hours prior to the first experimental trial participants were instructed to complete a self-report weighed food diary and a physical activity diary. Participants were asked to replicate these diary entries 24 h prior to the second and third experimental trials, where the extent of lifestyle replication was assessed via a written questionnaire. Participants were also provided with a food package for consumption on the evening before each experimental trial, the contents of which were standardised within a participant and provided an average of 437 kcal (∼58 % carbohydrate, ∼21 % fat, ∼21 % protein). To minimise intraindividual variability in physical activity on the morning of each experimental trial, participants were asked to record their means of commuting to the laboratory for their first trial and replicate this on the mornings of their subsequent experimental trials.

### Experimental protocol

Participants arrived at the laboratory at ∼07.00 h after an overnight fast (duration: 11:42 (sd 01:10) hh:mm; within-subject variation: 25 (sd 18) min), having refrained from strenuous physical activity and abstained from alcohol consumption for the preceding 24 h period. Upon arrival, body mass and height were measured, and body composition was assessed via Bioelectrical Impedance Analysis (TANITA SC-331S). Participants then rested in a semi-recumbent position with their forearms placed under a heated blanket to arterialise venous blood. After 10 min, a cannula (BD Venflon^TM^) connected to a three-way stopcock (BD Connecta^TM^) was inserted antegrade into an antecubital forearm vein, and a 15 ml arterialised blood sample was drawn. The cannula was then flushed with 5 ml sterile NaCl 0·9 % (BD PosiFlush^TM^) to maintain patency for repeated blood sampling (repeated at each blood sample). Participants were then asked to complete a series of 0–100 mm visual analogue scales to assess fasted-state appetite sensations: participants marked a line through the 100 mm scale to reflect how they felt in relation to the questions at the time of assessment. Four questions from this scale – ‘How hungry do you feel?’, ‘How full do you feel?’, ‘How satisfied do you feel?’ and ‘How much do you think you can eat?’ – were used to calculate a composite appetite score, as reported previously^(32)^. Following this, participants ingested 0·3 g·kg·body mass^–1^ of BLEND, PEA or WHEY, according to trial order randomisation, dissolved in 400 ml of water. On consumption initiation, a timer was started, where participants were asked to consume the beverage within 3 min. To ensure all residual protein was consumed, beverage containers were rinsed with a further 200 ml of water, which participants also consumed. Arterialised blood samples were drawn every 15 min during the first hour and every 30 min thereafter for the 4 h postprandial period. Appetite sensations were assessed via visual analogue scale at 5 min, 30 min and then hourly following protein ingestion for the remainder of the trial. At the hourly sampling timepoints, visual analogue scales were completed prior to arterialised blood sampling. Water intake was permitted *ad libitum* during the first 4 h trial and was recorded to ensure replication in subsequent trials. The cannula was removed following the 4 h postprandial period, and a buffet-style test meal was administered (04:09 (sd 00:01) hh:mm post-protein ingestion, within-subject variation: 2 (sd 1) min) to assess *ad libitum* energy intake. Participants were then free to leave the laboratory. Participants later returned to complete two further experimental trials, which were identical, except for the type of protein supplement they were asked to consume. At the end of their final trial, participants completed an exit questionnaire to determine the success of blinding to trial order.

### Supplemental beverages

The nutritional composition of the protein supplements was analysed by an independent third party (Premier Analytical Services; Table 2). Beverages were volume-matched and contained similar energy, carbohydrate, fat and fibre. Participants ingested 0·3 g·kg·body mass^–1^ of protein from BLEND, PEA or WHEY, which equated to a mean (sd) of 20·4 (sd 4·8) g protein for all treatments or 25·7 (sd 6·1) g (range 19·1–35·6 g), 25·5 (sd 6·0) g (range 18·9–35·3 g) and 24·9 (sd 5·9) g (range 18·5–34·5 g) of supplement material for BLEND, PEA and WHEY, respectively. The protein powders were all obtained from The Hut Group Ltd and were unflavoured, where participants were permitted a choice of three varieties of The Hut Group Ltd flavour drops (strawberry, vanilla or chocolate) to add to each beverage. Additional flavourings were standardised within participants and aimed to improve palatability and promote taste-matching. Beverages were served in identical opaque black shaker bottles to ensure participants were blind to beverage appearance.

**Table 2.** Nutritional composition of protein supplements

g/100 g	Pea protein isolate (PEA)	Pea-rice protein blend (BLEND)	Whey protein concentrate (WHEY)
Energy (kcal)	385·00	397·00	412·00
Protein	79·10	81·30	82·00
Fat	6·60	7·10	7·50
Carbohydrate	0·70	1·40	4·00
Fibre	3·20	1·20	0·00
Aspartic acid	8·99	8·74	8·25
Serine	4·18	4·31	3·85
Glutamic acid	12·50	13·60	13·33
Glycine	3·13	3·34	1·44
Histidine	1·78	1·82	1·39
Arginine	6·12	6·50	2·04
Threonine	2·69	2·79	5·27
Alanine	3·17	3·59	3·87
Proline	3·46	3·67	4·43
Cystine	0·67	0·99	1·77
Tyrosine	2·77	3·21	2·30
Valine	3·04	3·38	4·53
Methionine	0·80	1·19	1·67
Lysine	5·90	4·71	7·08
Isoleucine	2·57	2·61	4·78
Leucine	5·75	5·98	8·27
Phenylalanine	3·74	3·95	2·49
ΣTAA	71·26	74·38	76·76
ΣEAA	26·27	26·43	35·48
ΣNEAA	44·99	47·95	41·28

ΣTAA = summed total of total amino acids; ΣEAA = summed total of essential amino acids (His, Thr, Val, Met, Lys, Iso, Leu, Phe); ΣNEAA = summed total of non-essential amino acids.

### Blood sampling and analysis

Arterialised blood samples were collected into tubes containing anti-coagulant K^2^EDTA (BD Vacutainer®) and were placed on ice for 30 min before centrifugation at 4000 ***g*** for 10 min at 4°C. Plasma was aliquoted in duplicate and immediately transferred to −80°C for storage until further analysis. Plasma amino acid concentrations were analysed using reversed-phase ultra-performance LC-MS in collaboration with the Proteomics and Clinical Mass Spectrometry platform at the Research Institute of the McGill University Health Centre (Montreal, Quebec), as previously described^(28)^. Briefly, plasma amino acids were extracted via protein precipitation and derivatised with 6-aminoquinolyl-N-hydroxysuccinimidyl carbamate (AQC; Toronto Research Chemicals). Extracts were analysed using an Agilent 6460 triple quadrupole mass spectrometer coupled with an Agilent 1290 UPLC system (Agilent CA). Agilent MassHunter Data Acquisition (v. B.04·01) and Quantitative Analysis software (v. B.05·00) were used to perform data acquisition and sample quantification, respectively. Plasma glucose concentrations were measured in duplicate using an automated analyser (Rx Daytona, Randox Laboratories). Plasma concentrations of insulin, glucagon-like peptide-1 (total), peptide tyrosine tyrosine (total) and ghrelin (total) were measured in duplicate using ELISA kits (Mercodia; Sigma-Aldrich), according to manufacturer instructions, where all samples for a participant were measured on the same plate or run.

### Energy intake

Within-laboratory energy intake was assessed at each trial by the provision of a buffet-style *ad libitum* test meal comprising white bread, semi-skimmed milk, cornflakes, muesli, porridge oats, strawberry yogurt, margarine, strawberry jam, bananas and three varieties of cereal bar. To prevent any influence of external cues on eating behaviour, participants consumed the meal in isolation and were instructed to refrain from using their mobile phones throughout. Participants were instructed to ‘help themselves to the food items’ and to ‘eat as much or as little’ as they liked until comfortably full. Food items were weighed by the researcher before and after the test meal, where the weighted difference in food was recorded. Water intake was permitted *ad libitum* during the test meal. Within-laboratory energy intake was calculated using the following caloric values for each macronutrient: carbohydrate 3·75 kcal·g^–1^, fat 8·94 kcal·g^–1^, protein 4·02 kcal·g^–1(33)^.

### Statistical analysis

The required sample size was estimated using G * Power 3·1 software, based on a previous comparison of postprandial aminoacidemia following ingestion of wheat protein and a wheat-milk protein blend^(17)^. Plasma EAA 5 h incremental AUC (iAUC) was 72 (sd 9) *v*. 96 (sd 31) mmol·300 min·l^–1^ following wheat and wheat-milk blend ingestion, respectively. Based on this calculated effect size (*d* = 1·1), a two-tailed matched pairs design with nine participants would provide an 80 % chance (power) of detecting the stated effect with an *α*-level of 0·05. Descriptive statistics were calculated using Microsoft Excel. iAUC for postprandial metabolite and hormonal responses were calculated with the trapezoid method using the Time Series Response Analyser^(34)^. Figures were produced and statistical analysis performed in GraphPad Prism (v.9.5.1), where statistical significance was accepted at *P ≤* 0·05. Time-dependent variables were analysed using two-way repeated measures ANOVA or mixed-effects models (depending on missing data points) with *post hoc* Bonferroni correction. Time-independent variables were analysed using one-way repeated measures ANOVA or mixed-effects models (depending on missing data points) with *post hoc* Bonferroni correction. Data are presented as mean and 95 % CI unless otherwise stated.

## Results

### Plasma amino acid concentrations

**Fig. 2.**
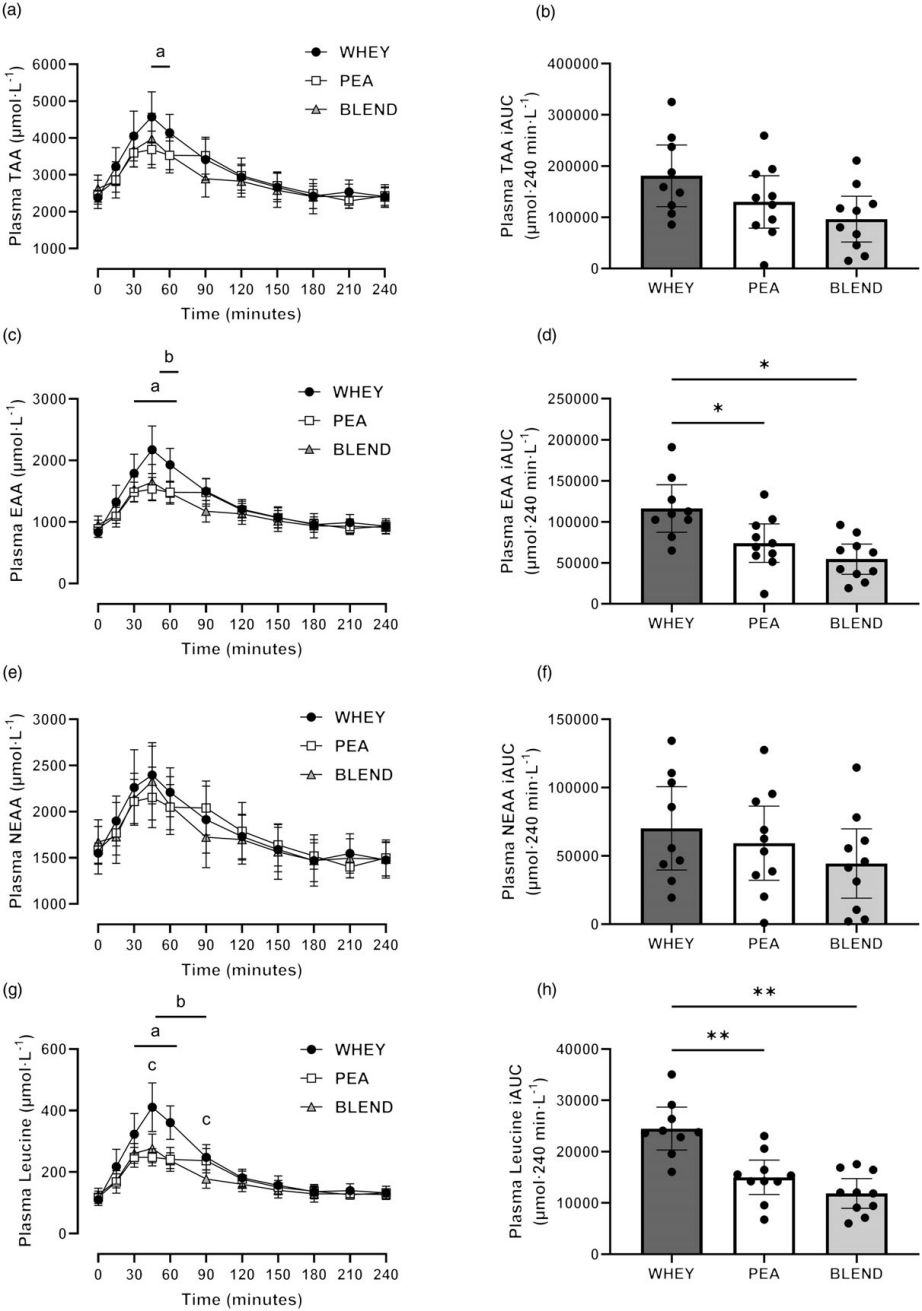
Postprandial plasma amino acid responses to ingestion of a pea-rice protein blend (BLEND), pea protein (PEA) and whey protein (WHEY) in healthy young adults. Time course and incremental area under the curves (iAUC) of plasma total amino acid (TAA) concentration (a), (b), plasma essential amino acid (EAA) concentration (c), (d), plasma non-essential amino acid (NEAA) concentration (e), (f) and plasma leucine concentration (g), (h). ^a^ denotes a statistically significant difference between WHEY and PEA (*P* < 0·05); ^b^ denotes a statistically significant difference between WHEY and BLEND (*P* < 0·05); ^c^ denotes a statistically significant difference between PEA and BLEND (*P* < 0·05); * *P* < 0·05; ** *P* < 0·01, respectively. *n* 10. Data presented as mean ± 95 % confidence intervals. EAA is the sum of His, Thr, Lys, Met, Val, Isl, Leu, Phe and Trp.

Plasma total amino acid (TAA) concentrations increased following protein ingestion (time effect: *P* < 0·001), with no main effect of trial detected (*P* = 0·118). However, a significant interaction effect was detected (*P* = 0·026; Fig. 2), where *post hoc* analysis revealed a significantly greater plasma TAA concentration following WHEY compared with PEA at 45 min (mean diff (95 % CI): 883 (82, 1683) μmol·l^–1^; *P* = 0·031) and 60 min (612 (70, 1153) μmol·l^–1^; *P* = 0·027). Peak plasma TAA concentrations were significantly greater following WHEY compared with PEA (807 (368, 1246) μmol·l^–1^; *P* = 0·001) and BLEND (735 (150, 1320) μmol·l^–1^; *P* = 0·015), with no significant differences between PEA and BLEND (*P* > 0·05). Time-to-peak TAA concentration did not differ between trials (*P* = 0·44). A significant main effect of trial was detected for plasma TAA iAUC (*P* = 0·028); however, differences did not remain following *post hoc* analysis.

There was a significant time (*P* < 0·001), trial (*P* = 0·004) and interaction effect (*P* = 0·001) for plasma EAA concentrations following protein ingestion (Fig. 2). Peak plasma EAA concentrations were significantly greater following WHEY compared with PEA (mean diff (95 % CI): 545 (253, 838) μmol·l^–1^; *P* = 0·001) and BLEND (521 (194, 849) μmol·l^–1^; *P* = 0·004), with no differences between PEA and BLEND (*P* > 0·05). No significant differences in time-to-peak plasma EAA concentration were detected between trials (*P* = 0·339). Plasma EAA iAUC was significantly greater following WHEY compared with PEA (42 244 (10 958, 73 530) μmol·240 min·l^–1^; *P* = 0·011) and BLEND (61 689 (13 786, 109 591) μmol·240 min·l^–1^; *P* = 0·014), with no significant differences between PEA and BLEND (*P* = 0·318).

Plasma non-essential amino acid (NEAA) concentrations increased following protein ingestion (time effect: *P* < 0·001), with no significant differences between trials (trial effect: *P* = 0·569; interaction effect: *P* = 0·233). There was a significant effect of trial for peak plasma NEAA concentration, where *post hoc* analysis revealed a significantly greater peak NEAA concentration following WHEY compared with PEA (mean diff (95 % CI): 254 (92, 416) μmol·l^–1^; *P* = 0·004). Time-to-peak plasma NEAA concentration did not differ between trials (*P* = 0·335), nor did plasma NEAA iAUC (*P* = 0·213).

There was a significant time, trial and interaction effect (*P* < 0·001 for all) for plasma leucine concentrations following protein ingestion (Fig. 2). Peak plasma leucine concentration was significantly greater following WHEY compared with PEA (mean diff (95 % CI): 147 (81, 212) μmol·l^–1^; *P* < 0·003) and BLEND (134 (59, 209) μmol·l^–1^; *P* = 0·002), with no significant differences between PEA and BLEND (*P* > 0·05). Time-to-peak plasma leucine concentration did not differ between trials (*P* = 0·567). Plasma leucine iAUC was significantly greater following WHEY compared with PEA (9503 (4078, 14 928) μmol·240 min·l^–1^; *P* = 0·002) and BLEND (12 651 (5101, 20 201) μmol·240 min·l^–1^; *P* = 0·003), with no significant differences between PEA and BLEND (*P* = 0·261).

**Fig. 3.**
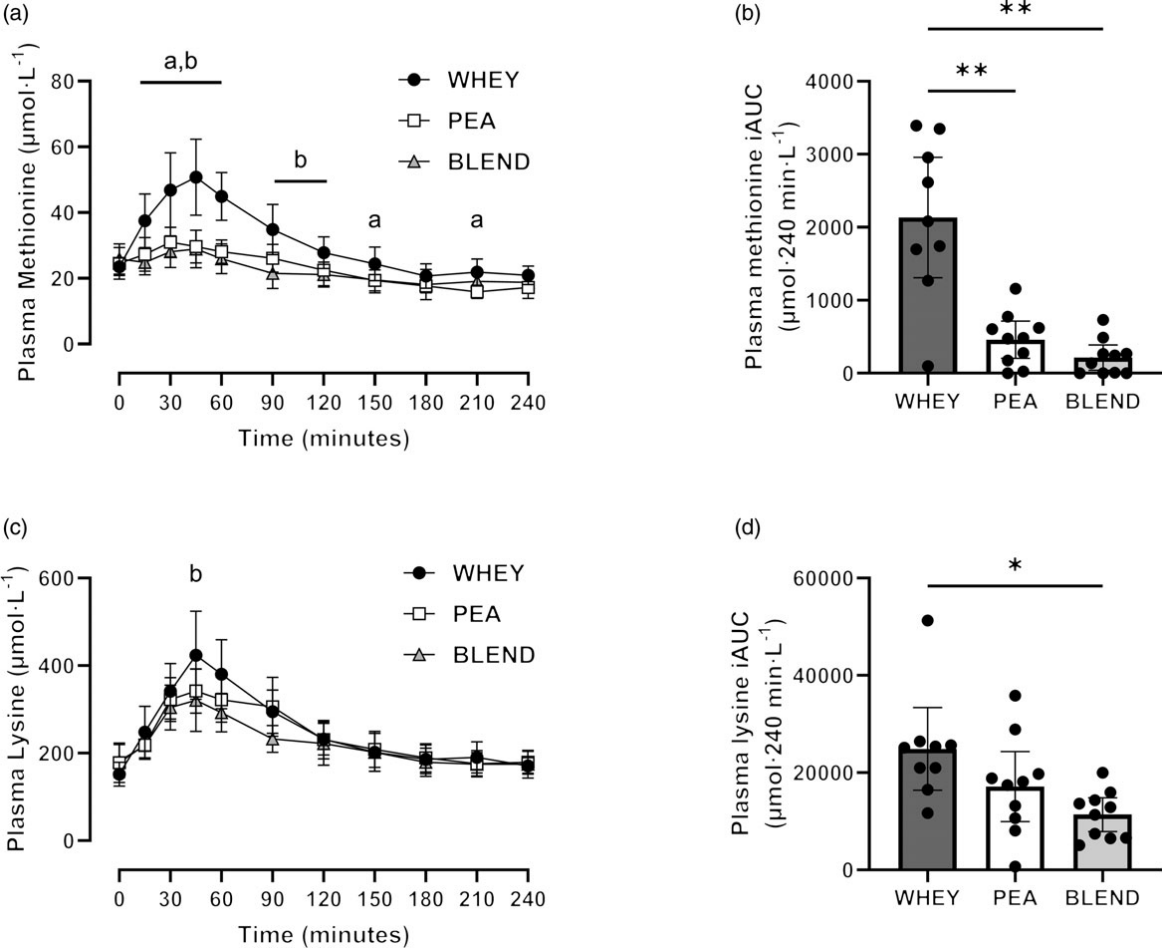
Postprandial plasma amino acid responses to ingestion of a pea-rice protein blend (BLEND), pea protein (PEA) and whey protein (WHEY) in healthy young adults. Time course and incremental iAUC of plasma methionine concentration (a), (b) and plasma lysine concentration (c), (d). ^a^ denotes a statistically significant difference between WHEY and PEA (*P* < 0·05); ^b^ denotes a statistically significant difference between WHEY and BLEND (*P* < 0·05); * *P* < 0·05; ** *P* < 0·01, respectively. *n* 10. Data presented as mean ± 95 % confidence intervals.

There was a significant time, trial and interaction effect (*P* < 0·001 for all) for plasma methionine concentrations following protein ingestion (Fig. 3). Peak plasma methionine concentration was significantly greater following WHEY compared with PEA (mean diff (95 % CI): 24 (11, 37) μmol·l^–1^; *P* = 0·001) and BLEND (27 (11, 42) μmol·l^–1^; *P* = 0·002), with no significant differences between PEA and BLEND (*P* > 0·05). Time-to-peak plasma methionine concentration did not differ between trials (*P* = 0·078). Plasma methionine iAUC was significantly greater following WHEY compared with PEA (1674 (690, 2658) μmol·240 min·l^–1^; *P* = 0·003) and BLEND (1919 (771, 3066) μmol·240 min·l^–1^; *P* = 0·003), with no significant differences between PEA and BLEND (*P* = 0·258).

Plasma lysine concentration increased following protein ingestion (time effect: *P* < 0·001), with no main effect of trial detected (*P* = 0·074; Fig. 3). However, a significant interaction effect was detected (*P* = 0·01), where *post hoc* analysis revealed a significantly greater plasma lysine concentration following WHEY compared with BLEND at 45 min (mean diff (95 % CI): 102 (9, 196) μmol·l^–1^; *P* = 0·033). Peak plasma lysine concentration was significantly lower following BLEND compared with both PEA (47 (18, 76) μmol·l^–1^; *P* = 0·003) and WHEY (132·0 (49, 215) μmol·l^–1^; *P* = 0·004), with no differences between PEA and WHEY (*P* = 0·067). Time-to-peak plasma lysine concentration did not differ between trials (*P* = 0·667). Plasma lysine iAUC was significantly greater following WHEY compared with BLEND (13 526 (769, 26 283) μmol·240 min·l^–1^; *P* = 0·038), with no differences between WHEY and PEA (*P* = 0·062) nor PEA and BLEND (*P* = 0·232).

### Plasma glucose and insulin concentrations

**Fig. 4.**
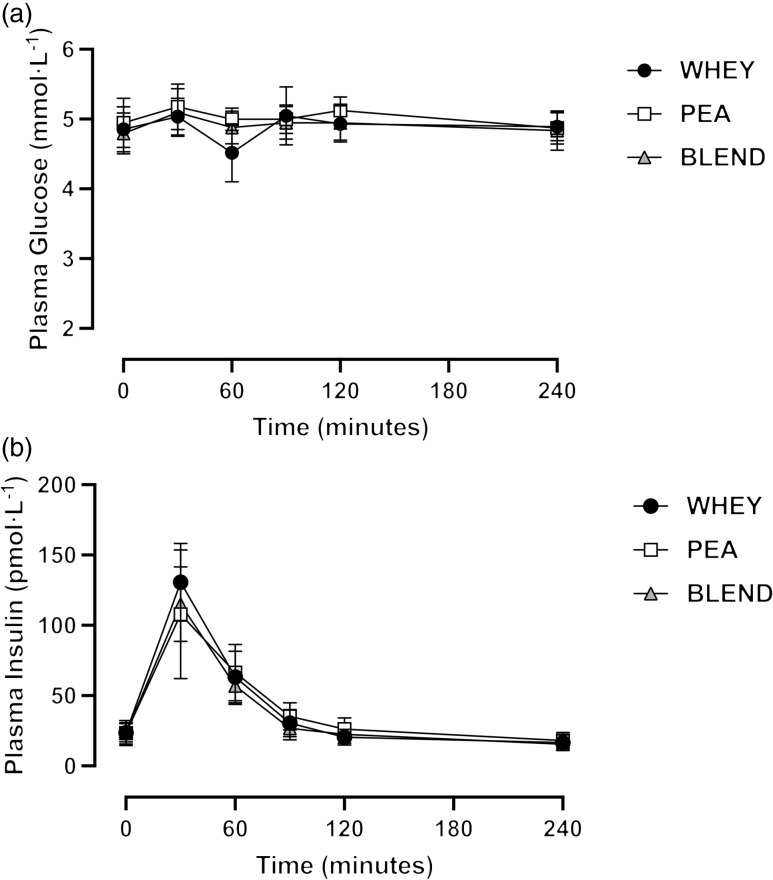
Postprandial plasma glucose (a) and insulin (b) concentrations following ingestion of a pea-rice protein blend (BLEND), pea protein (PEA) and whey protein (WHEY) in healthy young adults. *n* 10. Data presented as mean ± 95 % confidence intervals.

No statistically significant differences in plasma glucose concentrations were detected following ingestion of PEA, BLEND or WHEY (Fig. 4; time effect: *P* = 0·125; trial effect: *P* = 0·178; interaction effect: *P* = 0·165). Plasma insulin concentrations increased following protein ingestion (Fig. 4; time effect: *P* < 0·001), with no significant differences between trials (trial effect: *P* = 0·54; interaction effect: *P* = 0·21). Peak and time-to-peak plasma insulin concentrations did not differ between trials (*P* = 0·246 and *P* = 0·343, respectively), nor did plasma insulin iAUC (*P* = 0·494).

### Plasma appetite-regulatory hormone concentrations

**Fig. 5.**
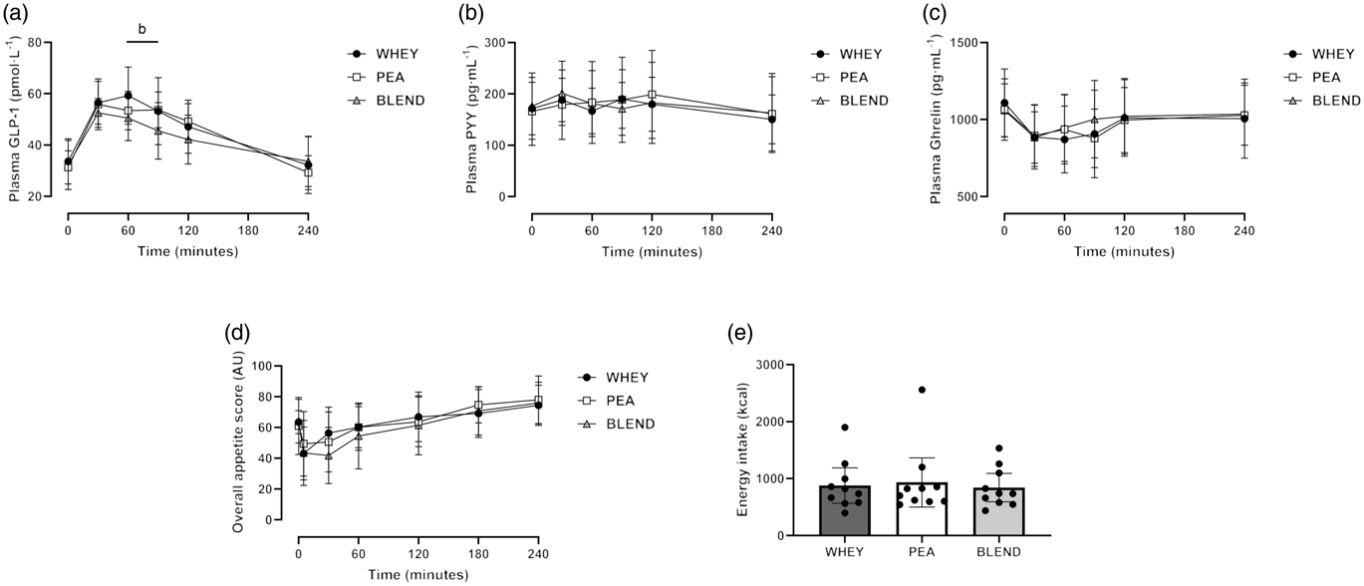
Postprandial appetite responses to ingestion of a pea-rice protein blend (BLEND), pea protein (PEA) and whey protein (WHEY) in healthy young adults. Time course of plasma total glucagon-like peptide-1 (GLP-1) (a), total peptide tyrosine tyrosine (PYY) (b) and total ghrelin (c) concentrations, overall appetite (d), calculated as composite score of hunger, prospective food consumption, fullness and satisfaction and test meal *ad libitum* energy intake (e). ^b^ denotes a statistically significant difference between WHEY and BLEND (*P* < 0·05). *n* 10. Data presented as mean ± 95 % confidence interval.

Plasma (total) ghrelin concentrations decreased following protein ingestion (time effect: *P* < 0·001), with no significant differences between trials (Fig. 5; time effect: *P* = 0·779; interaction effect: *P* = 0·135). Similarly, plasma (total) ghrelin total AUC did not differ between trials (*P* = 0·877). Plasma (total) glucagon-like peptide-1 increased over time following protein ingestion (Fig. 5; *P* < 0·001), with no significant main effect of trial detected (*P* = 0·204). However, a significant interaction effect was observed (*P* = 0·0321), where *post hoc* analysis revealed a significantly greater plasma (total) glucagon-like peptide-1 concentration following WHEY compared with BLEND at 60 min (mean diff (95 % CI): 9 (1, 17) μmol·l^–1^; *P* = 0·034) and 90 min (8 (0, 15) μmol·l^–1^; *P* = 0·045). Plasma (total) glucagon-like peptide-1 iAUC did not differ between trials (*P* = 0·062). There was a significant effect of time (*P* = 0·033), but neither the trial (*P* = 0·562) nor an interaction effect (*P* = 0·17) was detected for plasma (total) peptide tyrosine tyrosine concentrations, though the significant effect of time did not remain following *post hoc* analysis.

### Appetite sensations and energy intake

Subjective ratings of fasted-state and postprandial appetite sensations are displayed in Fig. 5. There were no significant between-trial differences in the ratings of hunger, satisfaction, fullness, prospective food consumption and desires for sweet and savoury foods (all *P* > 0·05). Overall appetite score was transiently altered by protein ingestion (Fig. 5; time effect: *P* < 0·001), with no significant differences between trials (trial effect: *P* = 0·575; interaction effect: *P* = 0·266). Similarly, there were no significant differences in overall appetite total AUC between trials (*P* = 0·541). *Ad libitum* energy intake during the buffet-style test meal did not differ between trials (Fig. 5; *P* = 0·529), nor did the consumption of carbohydrate, fat or protein (all *P* > 0·05).

### Standardisation and blinding

Protein beverages were correctly identified on only 27 % of occasions, where five of ten participants failed to identify a single beverage correctly. Trial order was correctly identified by only two of ten participants. The whey protein beverage was correctly identified on four occasions, whereas both the pea protein and pea-rice blend protein beverages were correctly identified on only two occasions each. *Ad libitum* energy, carbohydrate, fat and protein intake at the test meal did not display significant effects of trial order (*P* > 0·05 for all).

## Discussion

The present study compared postprandial plasma aminoacidemia and indices of appetite regulation following ingestion of a pea-rice protein blend (BLEND), pea protein isolate (PEA) and whey protein concentrate (WHEY) in healthy young adults. Postprandial (4 h) plasma availability of EAA and leucine following ingestion of this plant-derived protein blend was not significantly different compared with pea protein and was significantly lower compared with whey protein. Despite the apparent differences in postprandial plasma aminoacidemia between trials, plasma availability of gut-derived appetite hormones, subjective ratings of appetite sensations and *ad libitum* energy intake did not significantly differ between trials.

Owing to the growing concern regarding the long-term environmental impact of animal-derived protein production, investigations into the anabolic potential of non-animal-derived protein sources are timely. As plant-derived proteins are typically deficient in certain EAA^(11)^, plant-derived protein blends may offer a potential solution to bolster postprandial plasma amino acid availability^(20)^. Herein, we demonstrate for the first time that a plant-derived (pea-rice) protein blend did not augment 4 h postprandial plasma EAA availability above that of a single-source (pea-only) plant-derived protein. Despite specific methionine deficiency, pea protein may be regarded as a high-quality plant-derived protein^(11)^. Though the pea protein isolate used herein contained a high content of EAA, sufficiently meeting the requirements of the WHO/FAO/UNU^(35)^, low methionine content was still evident. In contrast, whilst the methionine content of the pea-rice protein blend was ∼1·5-fold higher than the pea protein isolate, the methionine content of the BLEND was still below the WHO/FAO/UNU^(35)^ requirements, and further, plasma methionine concentrations did not differ following ingestion of BLEND or PEA. As such, our results suggest plant-derived protein blends may not bolster postprandial essential aminoacidemia above that of a high-quality single-source plant protein isolate. Plant-blend proteins may be more efficacious at augmenting postprandial EAA bioavailability when compared with single-source plant proteins of lower quality. However, in the absence of sufficient evidence, future research should aim to characterise the postprandial amino acid and muscle anabolic responses to plant-blend *v*. low-quality single-source plant proteins, to further understand the context wherein plant-derived protein blends may hold the potential to optimise skeletal muscle remodelling.

As anticipated, the postprandial availability of EAA and leucine was greater following the ingestion of whey compared with pea protein and pea-rice blend protein, likely attributable to the ∼1·3-fold and ∼1·4-fold higher EAA and leucine content of the whey protein, respectively. In agreement, Pinckaers and colleagues^(21)^ reported a significantly lower 5 h plasma EAA availability following ingestion of a wheat-corn-pea protein blend compared with milk protein in healthy young adults. Similarly, van der Heijden *et al.*^(22)^ recently demonstrated a 44 % greater 4 h plasma EAA availability following ingestion of 32 g of whey protein, compared with a dose-matched pea-rice-canola protein blend. Collectively, this indicates inherent differences in the postprandial handling of animal- and plant-derived proteins, perhaps largely determined by protein digestion and absorption kinetics. Indeed, splanchnic retention of dietary protein-derived amino acids is thought to be greater following the ingestion of plant-derived protein sources compared with animal-derived protein sources^(36)^, which could explain the observed lower plasma EAA bioavailability following pea protein and pea-rice protein ingestion, compared with that of whey protein. Whether the observed difference in postprandial aminoacidemia between whey and plant-derived proteins results in divergent muscle anabolic responses is unclear. Evidence from acute experimental trials suggests that a certain threshold for the rise in postprandial essential aminoacidemia may be required to maximally stimulate rested and post-exercise muscle protein synthesis rates in healthy young individuals, beyond which any further increase has a negligible effect^(17,22,37)^. We suggest that longer-term intervention studies performed under free-living conditions would fully resolve the importance of postprandial essential aminoacidemia and leucinemia on muscle adaptive remodelling.

Despite the observed differences in postprandial aminoacidemia between the plant- and animal-derived protein beverages, indices of appetite regulation were not differentially altered according to protein source. Though the present study is the first to compare postprandial appetite responses following plant-blend and single-source plant-derived protein isolates, our findings corroborate previous studies reporting no significant effect of protein source on indices of acute appetite regulation^(26,31,38)^. It is possible that the degree of postprandial aminoacidemia reached following ingestion of all three protein treatments was sufficient to influence satiety and food intake similarly^(39)^, although non-protein or lower-protein treatments would be necessary to confirm this. Furthermore, we should highlight that the present study was solely statistically powered for our primary outcome (differences in postprandial plasma aminoacidemia between trials), and therefore, the study may have been underpowered to detect an effect of protein source on indices of appetite regulation. Omitting an assessment of beverage palatability herein is also an important limitation to consider since different sensory characteristics associated with each drink could have influenced our assessed measures of appetite. That said, if the drinks were deemed to be of different palatability, we expect that participants would have been able to correctly identify trial order, yet this was mostly not the case. We should also acknowledge that for our female participants, our decision to neither monitor the menstrual cycle phase nor characterise ovarian hormonal profiles to inform the scheduling of repeat trial visits may have contributed to these null findings, given the potential for menstrual cycle phase to influence indices of appetite regulation^(40)^. Alternatively, the duration between protein ingestion and the assessment of *ad libitum* energy intake in the present study may have prevented a significant effect of trial on energy intake from being detected^(41)^. Regardless, our findings may suggest that postprandial plasma amino acid concentrations do not primarily influence appetite regulation following ingestion of protein isolates from different sources.

In summary, the present study demonstrates for the first time that ingestion of a pea-rice protein blend does not augment postprandial plasma EAA availability above that of pea-only protein. Whilst this may raise questions around the efficacy of plant-derived protein blends as a means to augment postprandial aminoacidemia, our results are likely due to the high quality (and EAA content) of the pea-only protein comparator. As such, we speculate that when the EAA content of a single-source plant-derived protein is limiting, plant-derived protein blends may offer a pragmatic solution to bolster postprandial aminoacidemia, though further work is needed to ascertain this.
